# 4,7-Didehydro-neophysalin B Protects Rat Lung Epithelial Cells against Hydrogen Peroxide-Induced Oxidative Damage through Nrf2-Mediated Signaling Pathway

**DOI:** 10.1155/2022/4189083

**Published:** 2022-09-12

**Authors:** Qiu Zhong, Yaogui Sun, Ajab Khan, Jianhua Guo, Zhirui Wang, Na Sun, Hongquan Li

**Affiliations:** ^1^Shanxi Key Lab. for Modernization of TCVM, College of Veterinary Medicine, Shanxi Agricultural University, Taigu, 030801 Shanxi, China; ^2^Department of Veterinary Pathology, Faculty of Veterinary and Animal Sciences, The University of Agriculture, Dera Ismail Khan 29050, Khyber Pakhtunkhwa, Pakistan; ^3^USA Texas A&M University, College Station, TX, USA 77843; ^4^University of Colorado Denver Anschutz Medical Campus, RC2-6013, Mail Stop 8621 12700 E 19th Ave., Aurora, CO 80045, USA

## Abstract

The administration of 4,7-didehydro-neophysalin B is expected to be a promising strategy for mitigating oxidative stress in respiratory diseases. This study was aimed at investigating the efficacy of 4,7-didehydro-neophysalin B for apoptosis resistance of rat lung epithelial cells (RLE-6TN) to oxidative stress and evaluating its underlying mechanism of action. The RLE-6TN cells treated with hydrogen peroxide (H_2_O_2_) were divided into five groups, and 4,7-didehydro-neophysalin B was administered into it. To evaluate its mechanism of action, the expression of oxidative stress and apoptotic proteins was investigated. 4,7-Didehydro-neophysalin B significantly inhibited H_2_O_2_-induced RLE-6TN cell damage. It also activated the Nrf2 signaling pathway which was evident from the increased transcription of antioxidant responsive of KLF9, NQO1, Keap-1, and HO-1. Nrf2 was found to be a potential target of 4,7-didehydro-neophysalin B. The protein levels of Bcl-2 and Bcl-xL were increased while Bax and p53 were decreased significantly. Flow cytometry showed that 4,7-didehydro-neophysalin B protected RLE-6TN cells from apoptosis and has improved the oxidative damage. This study provided a promising evidence that 4,7-didehydro-neophysalin B can be a therapeutic option for oxidative stress in respiratory diseases.

## 1. Introduction

The respiratory epithelial cell damage is a key indicator of respiratory diseases. Investigation of respiratory epithelial cell injury and its underlying mechanism of action and development of new drugs against it are crucial steps to prevent and treat respiratory diseases. Hydrogen peroxide (H_2_O_2)_ is the main reactive oxygen species (ROS) involved in the regulation of redox reactions in biological activities through specific protein targets [1]. Recently, oxidative stress is considered as a key risk factor for respiratory diseases [2]; evidences showed that the causative factors of diseases including mycoplasma pneumonia are also linked with the oxidative stress [3–6]. Therefore, the active compounds with antioxidative properties tend to be the potential agents for the prevention of respiratory diseases.

In recent years, the use of active natural compounds against pathological conditions has gained considerable recognition. 4,7-Didehydro-neophysalin B and *Physalin* B are biologically active substances extracted from *Physalis alkekengi L. var. franchetii*. It has been confirmed that *Physalin* B has various pharmacological effects such as anti-inflammatory [7], antitumor [8], antibacterial [9], and immune regulation [10] verified by various bioassay both in vitro and in vivo. 4,7-Didehydro-neophysalin B is a new kind of *Physalin* B lacking two hydrogen atoms. There is evidence that *Physalin* B has efficient antioxidant activity [8]. However, the protective effect and mechanism of 4,7-didehydro-neophysalin B against H_2_O_2_-induced lung injury remained elusive.

Nuclear factor-erythroid 2-related factor-2 (Nrf2) has been identified as a key regulator of antioxidative, anti-inflammatory, and conjugation/detoxification proteins including NAD(P)H, quinine oxidoreductase 1 (NQO1), and heme oxygenase-1 (HO-1) [11]. After oxidative stress caused by H_2_O_2_ or other factors, the Nrf2 pathway is rapidly activated to eliminate intracellular ROS generation, thereby attenuating DNA damage induced by H_2_O_2_ to reduce risk of subsequent lung apoptosis [12].

Activation of antioxidative defense such as phase II enzyme expression is an effective way to protect cells against oxidative damage [13]. Nrf2 combined with antioxidant response element (ARE) system is one of the most important defensive signaling pathways to regulate the transcription activity of antioxidases [14]. The RLE-6TN cell line has been widely used to evaluate lung function due to their hypersensitivity to H_2_O_2_ [15, 16]. Although many bioactive compounds have been reported against oxidative stress, still there is no relevant study on the cytoprotective effect of 4,7-didehydro-neophysalin B on RLE-6TN cells.

In this study, H_2_O_2_ was used to establish the basic oxidative damage model so that we can investigate the effect of 4,7-didehydro-neophysalin B on the oxidative stress, cell apoptosis, and the role of Nrf2 signaling pathway. This study will promote the development of nutraceutical and functional food from *Physalis alkekengi L. var. franchetii* or its extracts for reducing the risk of oxidative stress-induced lung injury.

## 2. Materials and Methods

### 2.1. Preparation of 4,7-Didehydro-neophysalin B

Physalin was isolated from the stems of *Physalis alkekengi L. var. franchetii.* The plant was identified by Dr. Qiongming Xu from the College of Pharmaceutical Science, Soochow University. Physalin was purified, and the purity of Physalin is 72.39% by chromatography [17]. Physalin from the above reaction was pulverized, extracted with ethanol, loaded on a silica gel column, eluted, and purified with ethyl acetate. The obtained contents of 4,7-didehydro-neophysalin B were detected by chromatography.

### 2.2. Cytotoxicity of 4,7-Didehydro-neophysalin B

The RLE-6TN cells (ATCC® CRL-2300™, 4000 cells per well) were subcultured in 96-well plates. After different concentrations of 4,7-didehydro-neophysalin B (50, 25, 10, and 5 *μ*g/mL) treatments, cells were incubated with CCK-8 (CK04, Dojindo, Japan) solution (10%) for 1 h at 37°C. The resulting absorbance was measured at 450 nm by using a microplate reader (Tecan, Switzerland). The maximum nontoxic concentration (MNTC) value was calculated by Prism 6 software (San Diego, CA, USA) [18].

(1) $$\begin{array}{c} \text{Cell proliferation rate}=\frac{A_{450}\text{ of treatment group}}{A_{450}\text{ of blank group}}\times 100\%. \end{array}$$

### 2.3. Culture of RLE-6TN Cells

The RLE-6TN cell line was cultured in DMEM containing 15% fetal bovine serum, 2 mmol/L glutamine, 5 mmol/L sodium pyruvate, and 25 mmol/L HEPES, containing 100 UI/mL penicillin and 100 *μ*g/mL streptomycin, placed in an incubator and grown at 37°C, 5% CO_2_-saturated humidity. 0.25% trypsin-EDTA digested and passaged, and cells in logarithmic growth phase were used for experiments [19]. RLE-6TN cells were seeded in 96-well cell culture plates and incubated in a 5% CO_2_ humidified incubator for 24 h at 37°C. Various concentrations of H_2_O_2_ prepared in 2% medium (200, 100, 50, and 25 *μ*mol) were added and incubated again for 4, 8, 12, and 24 h. The cell proliferation rate of H_2_O_2_ was determined using the CCK-8 (CK04, Dojindo, Japan) kit. To develop a cellular oxidative damage model, H_2_O_2_ (100 *μ*mol) prepared in 2% medium was added to each well except the blank group and incubated for the next 12 h. Different concentrations of 4,7-didehydro-neophysalin B with low-dose group (2.5 *μ*g/mL), medium-dose group (5 *μ*g/mL), and high-dose group (10 *μ*g/mL) were added as treatment and incubated again for 24 h.

(2) $$\begin{array}{c} \text{Cell proliferation rate}=\frac{\;A_{450}\text{ of treatment group}}{A_{450}\text{ of blank group}}\times 100\%. \end{array}$$

### 2.4. Transfection

(1) Culture the RLE-6TN cells for 3 to 5 generations, select the cells with good growth condition, digest the cells with 0.25% trypsin, inoculate 2 × 10^5^ cells/well in 6-well plates, add 10% fetal bovine serum, culture in F12 medium (Gibco, USA) without double antibody for 24 h, and after the cell confluence reached 70%, transfection was carried out. (2) Mix 7.5 *μ*L of Lipofectamine 2000 (Life, USA) with 125 *μ*L of OPTI-MEM (Gibco, USA) medium. Take another EP tube and mix 6 *μ*L of siRNA (0.75 *μ*g) with 125 *μ*L of OPTI-MEM medium. This is the amount for one well in a 6-well plate. (3) Gently mix the two tubes of mixture in step 2, and let them act together at room temperature for 15 min. (4) The liquid after the joint action in step 3 was directly added to the original 6-well plate medium, and after 6 hours in a 37°C, 5% CO_2_ incubator, the medium was replaced and the subsequent experiments were carried out. For siRNA transfection, siRNA duplex targeting Nrf2 (sc-37030, Santa Cruz Biotechnology, Santa Cruz, CA, USA, primer shown in Table 1) were used. siRNA (sc-37007, Santa Cruz Biotechnology, Santa Cruz, CA, USA) was selected as a control to determine whether Nrf2's siRNA was successfully transfected.

### 2.5. Detection of Gene Expression in Tissues by RT-PCR

The total RNA was extracted from cells using TRIzol reagent, and the absorbance was measured at 260 and 280 nm by an ultraviolet spectrophotometer. The RNA content was calculated from the absorbance (*A*) value at a wavelength of 260 nm while the RNA purity was identified by the ratio of *A*_260_/*A*_280_. Reverse transcription of total RNA was performed with reverse transcriptase. The PCR primers (shown in Table 2) were designed and synthesized by KeyGEN BioTECH with GAPDH as endogenous control, and the PCR reactions were carried out according to the recommended conditions.

### 2.6. Flow Cytometry

After treating the cells with/without 100 *μ*mol of H_2_O_2_ for 12 h, different concentrations of 4,7-didehydro-neophysalin B (0, 2.5, 5, and 10 *μ*g/mL) were added to the rat lung epithelial cells RLE-6TN for 24 h. The cells were collected, washed twice with cold PBS, and centrifuged at 1000 rpm for 5 min, the supernatant was discarded, and the cells were made into a single cell suspension. 5 *μ*L of Annexin V⁃FITC and 5 *μ*L PI were added, mixed, and incubated for 15 min in the dark. Finally, 400 *μ*L of binding buffer was added and cell apoptosis was detected using flow cytometry.

### 2.7. Western Blot

RLE-6TN cells were homogenized with RIPA lysate (Solarbio, Beijing, China) containing protease inhibitor PMSF and phosphatase inhibitor (Solarbio). The nuclear and cytosolic proteins were extracted by a nuclear protein extraction kit (KeyGEN BioTECH, Jiangsu, China). The protein concentrations were measured by bicinchoninic acid (BCA) protein assay kit (Beyotime, Shanghai, China). 40 *μ*g equivalent protein samples were separated by SDS-PAGE gel electrophoresis and were transferred into a polyvinylidene fluoride (PVDF) membrane. The membranes were incubated with appropriate primary and HRP-conjugated secondary antibodies. Chemiluminescence was visualized with an enhanced chemiluminescence kit (BOSTER, Wuhan, China). Glyceraldehyde-3-phosphate dehydrogenase (GAPDH) protein (Sanying, Wuhan, China) and the TATA-binding protein (TBP) (Sanying, Wuhan, China) were used as loading control. The antibodies for Nrf2, heme oxygenase-1 (HO-1), nicotinamide adenine dinucleotide phosphatase:quinone-acceptor 1 (NQO1), Krueppel-like factor 9 (KLF9), Keap-1, p53, Bcl-2-associated X protein (Bax), B cell lymphoma gene 2 (Bcl-2), and B cell lymphoma-extra large (Bcl-xL) were purchased from Abcam (UK). All secondary antibodies were from Sanying (Wuhan, China). Protein expression was analyzed by scanning densitometry using ImageJ software (USA).

To further explain the molecular mechanism by which 4,7-didehydro-neophysalin B treated H_2_O_2_-induced oxidative damage in RLE-6TN cells, western blot was performed. Nrf2's siRNA was generated to inhibit Nrf2 expression, and the inhibitory efficiency was verified using western blot. Nrf2's siRNA was transfected into RLE-6TN cells with or without H_2_O_2_ treatment. Then, 10 *μ*g/mL 4,7-didehydro-neophysalin B was administrated for treatment and the expression of Nrf2 was explored using western blotting.

### 2.8. Statistical Analysis

Statistical analysis was performed by one-way analysis of variance with Tukey's test post hoc comparisons and Student's *t*-test when comparing between 2 groups using SPSS 19.0 software (USA). The data were presented as the mean ± SEM. Values with *p* < 0.05 was considered statistically significant.

## 3. Results

### 3.1. Structure and Content of 4,7-Didehydro-neophysalin B

**Figure 1:**
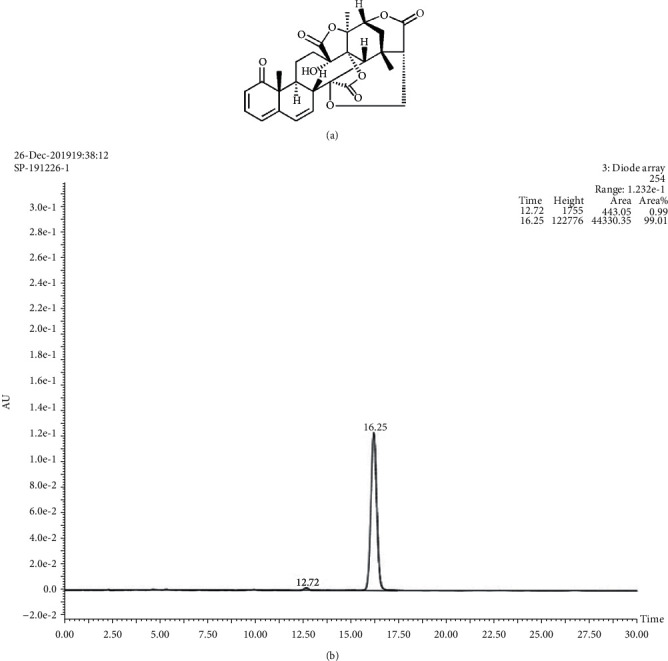
(a) The structure of 4,7-didehydro-neophysalin B. (b) The contents of 4,7-didehydro-neophysalin B.

The structure and contents of 4,7-didehydro-neophysalin B are shown in Figures 1(a) and 1(b). The chemical formula of 4,7-didehydro-neophysalin B is C_28_H_28_O_9_, and its purity is 99.01%.

### 3.2. The Cytotoxicity of 4,7-Didehydro-neophysalin B

**Figure 2:**
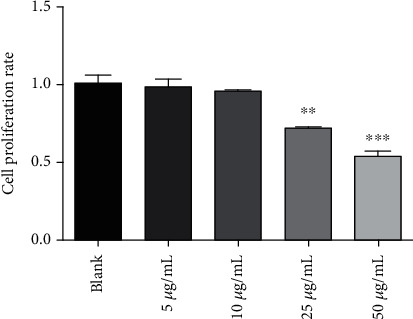
Cell viability (CCK-8) assay results of RLE-6TN cells: 4,7-didehydro-neophysalin B-induced cell proliferation rate. Compared with the blank group, ^∗^*p* < 0.05, ^∗∗^*p* < 0.01, and ^∗∗∗^*p* < 0.001.

The maximum nontoxic concentration of 4,7-didehydro-neophysalin B in RLE-6TN was investigated. Compared with the blank group, RLE-6TN cells treated with 25 *μ*g/mL 4,7-didehydro-neophysalin B for 24 h showed significant reduction in cell viability (74.87 ± 1.54%, *p* = 0.008 < 0.01) while 10 *μ*g/mL 4,7-didehydro-neophysalin B had negligible effect on cell viability (93.6 ± 1.03%, *p* = 0.07 > 0.05) (Figure 2). Therefore, we choose 10 *μ*g/mL 4,7-didehydro-neophysalin B as the maximum nontoxic concentration.

### 3.3. The Effect of 4,7-Didehydro-neophysalin B on RLE-6TN Pretreated with H_2_O_2_

**Figure 3:**
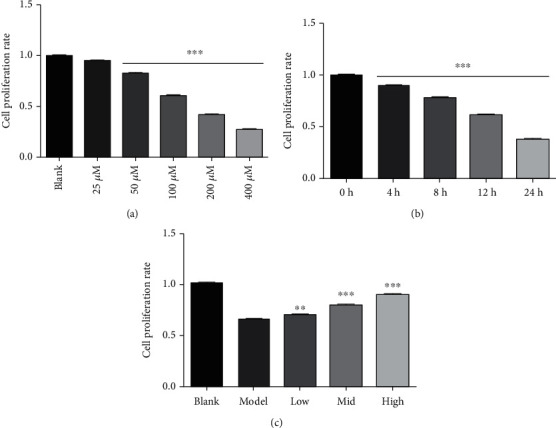
Cell viability (CCK-8) assay results of RLE-6TN cells. (a) H_2_O_2_-induced cell proliferation rate (concentration-dependent); (b) H_2_O_2_-induced cell proliferation rate (time-dependent); (c) 4,7-didehydro-neophysalin B treatment of the H_2_O_2_-challenged cells. Low: 2.5 *μ*g/mL 4,7-didehydro-neophysalin B-treated RLE-6TN cell group; mid: 5 *μ*g/mL 4,7-didehydro-neophysalin B-treated RLE-6TN cell group; high: 10 *μ*g/mL 4,7-didehydro-neophysalin B-treated RLE-6TN cell group; the same as below. Compared with the blank group, ^∗^*p* < 0.05, ^∗∗^*p* < 0.01, and ^∗∗∗^*p* < 0.001. Compared with the model group, ^#^*p* < 0.05, ^##^*p* < 0.01, and ^###^*p* < 0.001.

Compared with the blank group, the working concentration for H_2_O_2_ in RLE-6TN cells treated with different concentrations was determined. Results showed that 100 *μ*mol H_2_O_2_ treated for 12 h significantly reduced (59.87 ± 1.32%, *p* = 0.0004 < 0.001) while 50 *μ*mol H_2_O_2_ showed less effect on the cell viability (80.37 ± 1.96%, *p* = 0.0007 < 0.001). The mortality of cells treated with 200 *μ*mol of H_2_O_2_ was too high (41.28 ± 2.84%, *p* = 0.0003 < 0.001) as shown in Figure 3(a). Therefore, 100 *μ*mol H_2_O_2_ was selected as a working concentration.

Compared with the blank group, the working time for 100 *μ*mol H_2_O_2_ in RLE-6TN cells treated with different times was determined. The results showed that RLE-6TN cells treated with 100 *μ*mol H_2_O_2_ for 24, 12, 8, and 4 h treatment all had significant effect on cell viability, but the RLE-6TN cells treated with 100 *μ*mol H_2_O_2_ for 24 (37.84 ± 1.62%, *p* = 0.0005 < 0.001), 8 (76.62 ± 1.45%, *p* = 0.0006 < 0.001), and 4 (87.61 ± 2.33%, *p* = 0.0009 < 0.001) hours were too high or low on cell viability, as shown in Figure 3(b). Therefore, 12 h (68.88 ± 0.95%, *p* = 0.0002 < 0.001) was selected as a working time.

**Figure 4:**
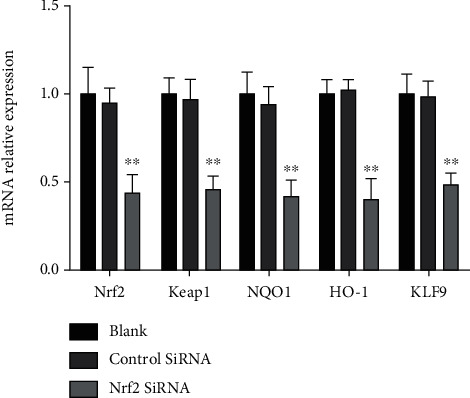
The mRNA expression of Nrf2 and its downstream genes. Compared with the blank group, ^∗^*p* < 0.05 and ^∗∗^*p* < 0.01.

Conclusively, 100 *μ*mol of H_2_O_2_ was used to treat RLE-6TN for 12 h, followed by the addition of 4,7-didehydro-neophysalin B to examine the effect on cell viability. As shown in Figure 3(c), 4,7-didehydro-neophysalin B had significantly reversed the cell viability caused by H_2_O_2_ in a dose-dependent manner in which 10 *μ*g/mL 4,7-didehydro-neophysalin B showed the strongest effect (84.93 ± 1.86%, *p* = 0.0007 < 0.001).

### 3.4. Expressions of Nrf2 in RLE-6TN under the Intervention of Nrf2's siRNA

The mRNA expression of Nrf2 and its downstream genes was detected by RT-PCR. The *A*_260_/*A*_280_ ratio of the RNA samples was 1.8-2.0. Nrf2's siRNA treatment significantly reduced the mRNA expression of Nrf2 and its downstream genes compared with the control siRNA which have shown negligible effect on the mRNA expression of Nrf2 and its downstream genes, as shown in Figure 4.

### 3.5. 4,7-Didehydro-neophysalin B Mitigates H_2_O_2_-Induced Apoptosis in RLE-6TN

**Figure 5:**
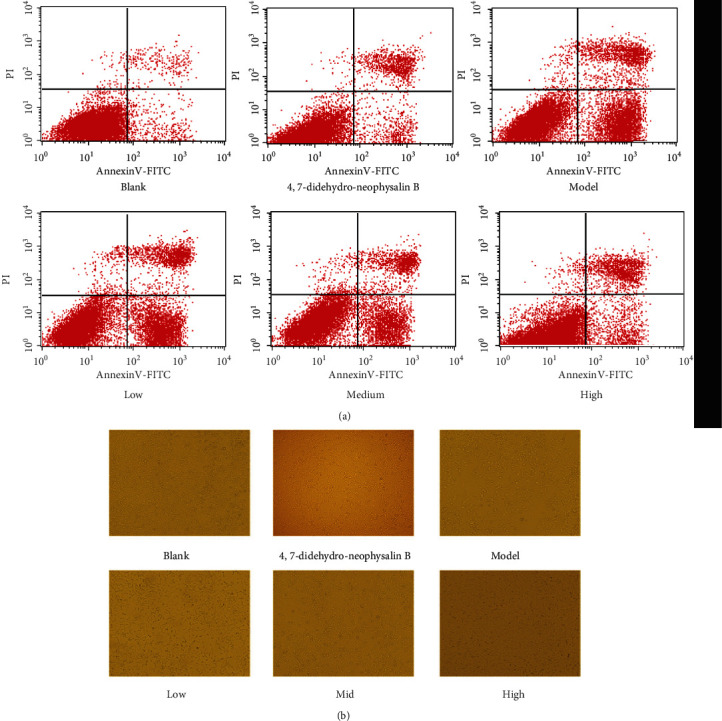
(a) The effect of 4,7-didehydro-neophysalin B on H_2_O_2_-induced apoptosis in RLE-6TN cells. (b) The microscope images of H_2_O_2_-induced cells after treatment with 4,7-didehydro-neophysalin B.

We investigated the effect of 4,7-didehydro-neophysalin B on H_2_O_2_-induced cell apoptosis by flow cytometry. Compared with the blank group, H_2_O_2_ caused a significant increase in RLE-6TN cell apoptosis while only 4,7-didehydro-neophysalin B treatment showed no significant effect on cell viability. Similarly, 4,7-didehydro-neophysalin B treatment dramatically mitigated H_2_O_2_-induced apoptosis. These results elucidated that 4,7-didehydro-neophysalin B had only a significant effect on H_2_O_2_-induced early apoptosis but had no significant effect on late apoptosis (early apoptosis: 4,7-didehydro-neophysalin B group 7.98 ± 1.15%, *p* = 0.07 > 0.05; model group 37.48 ± 3.84%, *p* = 0.0005 < 0.001; low-dose group 29.83 ± 3.68%, *p* = 0.0007 < 0.001; medium-dose group 21.44 ± 2.99%, *p* = 0.0008 < 0.001; high-dose group 15.35 ± 1.68%, *p* = 0.03 < 0.05, compared with the blank group) (late apoptosis: 4,7-didehydro-neophysalin B group 13.32 ± 2.47%, *p* = 0.008 < 0.01; model group 24.49 ± 2.96%, *p* = 0.006 < 0.01; low-dose group 17.92 ± 2.86%, *p* = 0.007 < 0.01; medium-dose group 15.58 ± 3.15%, *p* = 0.008 < 0.01; high-dose group 19.44 ± 3.57%, *p* = 0.006 < 0.01, compared with the blank group), as shown in Figure 5(a). The microscopic images of cells after treatment are shown in Figure 5(b).

### 3.6. The Effect of 4,7-Didehydro-neophysalin B on Nrf2-Dependent Signaling Pathway in H_2_O_2_-Induced Oxidative Damage

**Figure 6:**
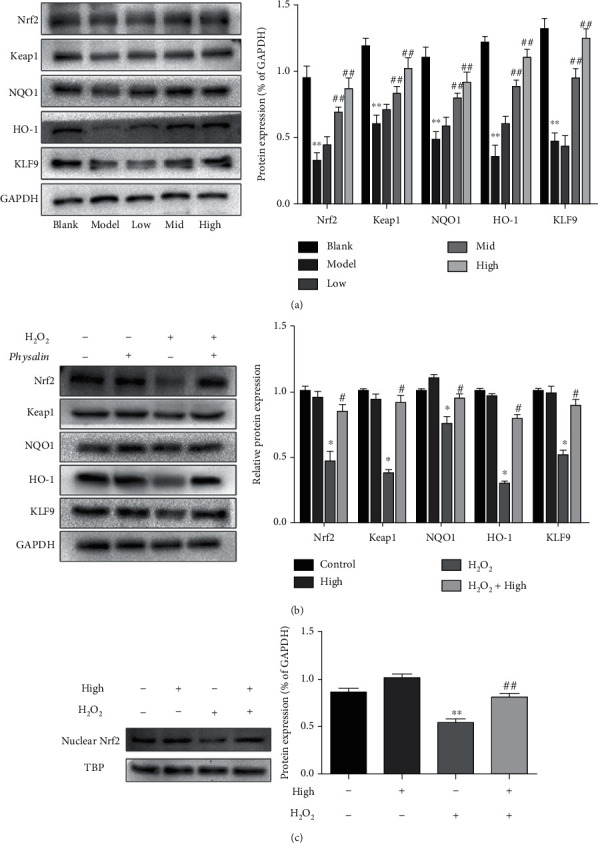
Western blot analysis of the Nrf2 signaling pathway. (a) After being added with different concentrations of 4,7-didehydro-neophysalin B, Nrf2 pathway-related protein expression in RLE-6TN cells and its quantitative band intensity analysis. (b) Effect of 4,7-didehydro-neophysalin B on the expression of Nrf2 pathway-related proteins and the quantitative band intensity analysis. (c) Expression of Nrf2 protein in the nucleus and its quantitative band intensity analysis. Compared with the blank group, ^∗^*p* < 0.05, ^∗∗^*p* < 0.01, and ^∗∗∗^*p* < 0.001. Compared with the H_2_O_2_+0% 4,7-didehydro-neophysalin B group, ^#^*p* < 0.05, ^##^*p* < 0.01, and ^###^*p* < 0.001.

Nrf2 signaling pathway proteins were detected by western blotting. In vitro treatment of H_2_O_2_ significantly reduced the Nrf2, NQO1, Keap1, HO-1, and KLF9 protein expressions. However, 4,7-didehydro-neophysalin B treatment has significantly reversed these effects induced by H_2_O_2_ with 10 *μ*g/mL 4,7-didehydro-neophysalin B showing the strongest effect as shown in Figure 6(a). The addition of 4,7-didehydro-neophysalin B alone showed no effect on the protein expression of Nrf2, NQO1, Keap1, HO-1, and KLF9 in RLE-6TN as shown in Figure 6(b). H_2_O_2_ had markedly reduced the expression of nuclear Nrf2 which was significantly reversed by 4,7-didehydro-neophysalin B as shown in Figure 6(c).

### 3.7. Knockdown of Nrf2 Declines the Treatment Effect of *Physalin* B

**Figure 7:**
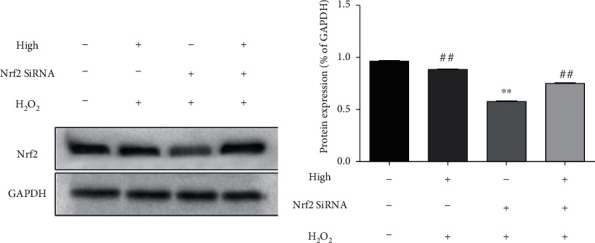
After using Nrf2 siRNA to knock down the expression of Nrf2 protein, effect of 4,7-didehydro-neophysalin B on the expression of Nrf2 pathway-related proteins and their quantitative band intensity analysis. Compared with the blank group, ^∗^*p* < 0.05 and ^∗∗^*p* < 0.01. Compared with the H_2_O_2_+0% 4,7-didehydro-neophysalin B group, ^#^*p* < 0.05 and ^##^*p* < 0.01.

The Nrf2's siRNA treatment significantly reduced the expression of Nrf2 as shown in Figure 7. Compared with the model group, the treatment effect of 4,7-didehydro-neophysalin B in the Nrf2's siRNA treatment group was significantly reduced.

### 3.8. Effects of 4,7-Didehydro-neophysalin B on Bcl-2 Family and p53 Proteins

**Figure 8:**
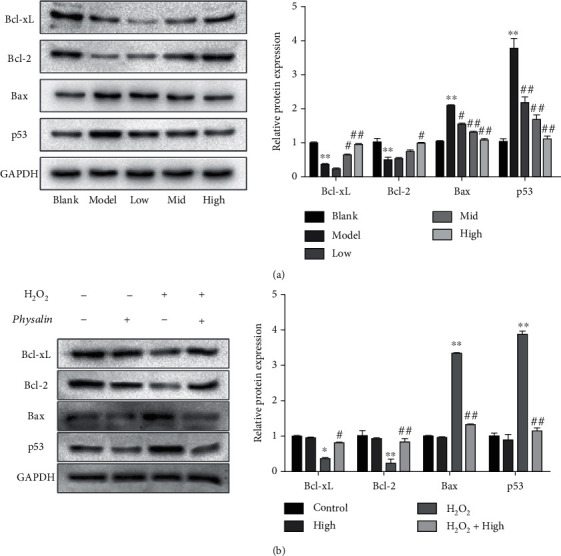
Western blot analysis of p53 pathway protein. (a) Post addition of different 4,7-didehydro-neophysalin B concentrations, p53 pathway-related protein expression in RLE-6TN cells and their quantitative band intensity analysis. (b) Effect of 4,7-didehydro-neophysalin B on the expression of p53 pathway-related proteins and the quantitative band intensity analysis. Compared with the blank group, ^∗^*p* < 0.05 and ^∗∗^*p* < 0.01, and with the H_2_O_2_+0% 4,7-didehydro-neophysalin B group, ^#^*p* < 0.05 and ^##^*p* < 0.01.

The molecular basis of a protective effect of 4,7-didehydro-neophysalin B against H_2_O_2_-induced cell apoptosis was investigated. H_2_O_2_ treatment decreased the expression of antiapoptotic Bcl-2 and Bcl-xL proteins in RLE-6TN cells. In H_2_O_2_-treated cells, 4,7-didehydro-neophysalin B administration significantly induced the expression of Bcl-2 and Bcl-xL. The expression of proapoptotic protein Bax was significantly increased post H_2_O_2_ treatment which was significantly reduced by 4,7-didehydro-neophysalin B treatment. Compared with the control group, p53 expression was also increased post H_2_O_2_ treatment. 4,7-Didehydro-neophysalin B attenuated H_2_O_2_-induced p53 expression with the strongest effect of 10 *μ*g/mL 4,7-didehydro-neophysalin B as shown in Figure 8(a) (Bcl-xL expression: blank 1.0 ± 0.14, control 0.37 ± 0.05, *p* = 0.004 < 0.01 compared with the blank group; low 0.28 ± 0.07, mid 0.59 ± 0.09, *p* = 0.03 < 0.05 compared with the control group; high group 0.98 ± 0.05, *p* = 0.11 compared with the control group; Bcl-2 expression: blank 1.0 ± 0.16, control 0.48 ± 0.35, *p* = 0.005 < 0.01 compared with the model group; low 0.53 ± 0.005, mid 0.59 ± 0.77, *p* = 0.04 < 0.05 compared with the control group; high group 0.98 ± 0.05, *p* = 0.11 < 0.01 compared with the control group). The addition of 4,7-didehydro-neophysalin B alone showed no effect on the protein expression of Bcl-2, Bcl-xL, Bax, and p53 in RLE-6TN as shown in Figure 8(b).

## 4. Discussion

A variety of chemicals and environmental factors causes oxidative stress in the human body which damages and induces a variety of diseases. H_2_O_2_-induced RLE-6TN cell damage is a classical model of oxidative stress injury. Studies have shown that the damage caused by oxidative stress is mediated by reactive oxygen species which plays an important role in neurodegenerative diseases [20, 21]. H_2_O_2_ produced in the body during metabolism is equivalent to reactive oxygen species and can aggravate the body oxidative stress injury [22, 23]. The results of this study showed that H_2_O_2_ induced oxidative damage and apoptosis in RLE-6TN cells which was reduced by 4,7-didehydro-neophysalin B treatment as shown in Figure 2(c). This study demonstrated that 4,7-didehydro-neophysalin B possessed therapeutic effects in the form of antioxidant activities against H_2_O_2_-induced oxidative stress injury.

When the cellular level of ROS exceeds the body's antioxidant capacity, it induces oxidative stress leaving the cells in a redox state by producing peroxides and free radicals [24–26]. Mitochondrial dysfunction and DNA damage are caused by the accumulation of reactive oxygen species which mediate and accelerate apoptosis [27–30]. DNA damage causes activation of p53which serves as a major mediator of cellular stress [31]. Our results (Figure 7) indicated p53-mediated apoptosis in H_2_O_2_-induced lung injury. p53 along with the members of Bcl-2 family proteins regulates protein–protein interactions and causes activation of Bax which promotes mitochondrial membrane permeability and hence induces apoptosis [32]. In this study, 4,7-didehydro-neophysalin B suppressed p53, decreased the level of proapoptotic member Bax, and increased the level of prosurvival members Bcl-2 and Bcl-xL. Moreover, the results of apoptotic index (Figure 2) and cell viability (Figure 4) of RLE-6TN were consistent with the observed effects on Bcl-2 family proteins. These findings suggest that 4,7-didehydro-neophysalin B has a pivotal role in regulating apoptosis and inhibiting oxidative stress-induced cell death. It is also noteworthy that some studies showed that NQO1 stabilizes the tumor suppressor p53 [33]. HO-1 upregulates Bcl-2 and Bcl-xL expressions [34] which are downstream proteins of Nrf2. A possible explanation is that 4,7-didehydro-neophysalin B protects lung injury induced by H_2_O_2_ via activating the Nrf2 pathway.

The transcription factor Nrf2 plays an important role in protection against oxidative damage [35, 36]. Nrf2 senses the presence of oxidative stress and regulates transcription of genes encoding cytoprotective enzymes and other proteins crucial for maintaining cellular homeostasis. Under physiological conditions, the Nrf2 inhibitor, Keap-1, which is a negative regulator of Nrf2 binds to Nrf2 and retains Nrf2 in the cytoplasm [37]. During oxidative stress, Nrf2 dissociates from Keap1, translocates into the nucleus, and activates ARE-dependent gene expression including transcription of target genes NQO-1 and HO-1 [38], thereby improving the antioxidant capacity in the body [39, 40]. Accumulation of Nrf2 in the nucleus is a necessary condition which is closely related to the induction of cellular defense genes [41]. The increase in cell death caused by H_2_O_2_ might be attributed to the insufficient ROS removal caused by the failure of Nrf2 activation. However, Figure 5 shows that 4,7-didehydro-neophysalin B activates the antioxidant pathway affecting both Nrf2 gene levels and expression of its target proteins. Therefore, this study suggested that Nrf2 activation was required for protection of the lungs by 4,7-didehydro-neophysalin B from H_2_O_2_-mediated cell death.

## 5. Conclusions

In conclusion, our study demonstrated that *Physalin* B attenuated H_2_O_2_-induced lung injury by regulating the Nrf2/P53 signaling pathway. *Physalin* B with great therapeutical potential can be used as an antioxidant agent. This study provided beneficial evidences for the application of *Physalin* B supplementation as an alternative treatment strategy for lung injury.

## Figures and Tables

**Table 1:** Nrf2 siRNA: a pool of 3 different siRNA duplexes.

siRNA	Primer (5′→3′)
Nrf2 A	Sense: GCAUGCUACGUGAUGAAGAtt Antisense: UCUUCAUCACGUAGCAUGCtt
Nrf2 B	Sense: CUCCUACUGUGAUGUGAAAtt
	Antisense: UUUCACAUCACAGUAGGAGtt
Nrf2 C	Sense: GUGUCAGUAUGUUGAAUCAtt
	Antisense: UGAUUCAACAUACUGACACtt

**Table 2:** Primers used in RT-PCR.

Gene	Primer (5′→3′)
Nrf2	Forward: GAGAGCCCAGTCTTCATTGC Reverse: TTGGCTTCTGGACTTGGAAC
Keap1	Forward: TTCAAGGCCATGTTCACCAA
	Reverse: TGGATACCCTCAATGGACACC
NQO1	Forward: GGAGAGTTTGCTTACACTTACGC
	Reverse: AGTGGTGATGGAAAGCACTGCCTTC
HO-1	Forward: CGCCTTCCTGCTCAACATT
	Reverse: TGTGTTCCTCTGTCAGCATCAC
KLF9	Forward: GGGAAACCTCCGAAAA
	Reverse: CGTTCACCTGTATGCACTGTA
GAPDH	Forward: GGAGATTACTGCCCTGGCTC Reverse: GACTCATCGTACTCCT

RT-PCR was performed on the ABI PRISM® 7500 real-time PCR analyzer (Applied Biosystems, Foster City, CA, USA) using the SYBR® Premix Ex Taq™ RT-PCR Kit. The relative mRNA expression was calculated by means of 2^−*ΔΔ*Ct^ and was normalized to the mean mRNA expressions of GAPDH. The results were calculated with the following formulae: ratio = 2^−ΔΔCt^, ΔΔCt = (Ct_target_ − Ct_GAPDH_)_Sample_ − (Ct_target_ − Ct_GAPDH_)_Control_.

## Data Availability

The original contributions presented in the study are included in the article; further inquiries can be directed to the corresponding author.

## Abbreviations

H_2_O_2_: Hydrogen peroxide
ROS: Reactive oxygen species
Nrf2: Nuclear translocation of erythroid-2-related factor 2
PVDF: Polyvinylidene fluoride
GAPDH: Glyceraldehyde-3-phosphate dehydrogenase
ARE: Antioxidant response element
TBP: TATA-binding protein
HO-1: Heme oxygenase-1
NQO1: Nicotinamide adenine dinucleotide phosphatase:quinone-acceptor 1
KLF9: Krueppel-like factor 9
Keap-1: Kelch-like ECH-associated protein
Bax: Bcl-2-associated X protein
Bcl-2: B cell lymphoma gene 2
Bcl-xL: B cell lymphoma-extra large
SEM: Standard errors of the mean.
